# Effect factors related to a high probability of hemodialysis independence in newly diagnosed multiple myeloma patients requiring hemodialysis

**DOI:** 10.1002/jcla.23057

**Published:** 2019-10-30

**Authors:** Jia Song, Fengjuan Jiang, Hui Liu, Kai Ding, Yue Ren, Lijuan Li, Guojin Wang, Zonghong Shao, Rong Fu

**Affiliations:** ^1^ Department of Hematology Tianjin Medical University General Hospital Tianjin China

**Keywords:** dialysis independence, effect factors, hemodialysis, multiple myeloma, renal failure

## Abstract

**Background:**

Renal failure is a severe complication of symptomatic myeloma, related to higher mortality. Recovery from dialysis dependence can lead to enormous survival benefits. We investigated the effect factors for probability of dialysis independence.

**Methods:**

Retrospective data on 45 newly diagnosed MM (NDMM) patients with serious renal impairment and requiring hemodialysis were analyzed. The statistical methods including logistic regression analysis, Kaplan‐Meier survival curves, the log‐rank test and the Cox proportional hazards model for survival analysis were used in our study.

**Results:**

Twenty‐two of the 45 patients, who were on hemodialysis at diagnosis, became dialysis independence. In the logistic regression analysis, serum level of β2‐microglobulin, kidney disease history, involved free light chain, and achieving at least VGPR were significantly associated with reversibility from dialysis dependence. In addition, achieving hemodialysis discontinuation was related to better survival. The multivariate analyses demonstrated that reversibility from dialysis dependence, proteinuria < 3.5 g/24 h, and achieving at least VGPR were significantly associated with OS among NDMM patients requiring hemodialysis.

**Conclusion:**

Lower serum level of β2‐microglobulin and lower level of free light chain at diagnosis, achieving at least VGPR, and shorter kidney disease history are related to a high probability of dialysis independence in NDMM patients with serious renal failure requiring dialysis.

## 1 INTRODUCTION

Multiple myeloma (MM) is a hematologic malignancy because of the uncontrolled proliferation of plasma cells in the bone marrow (BM). Renal impairment is a common complication of multiple myeloma (MM), with 20% to 50% of patients with an upper serum creatinine comparing to normal at diagnosis.^1^, ^2^, ^3^ In addition, 10% of MM patients can occur acute and severe renal failure (RF) requiring hemodialysis.^4^ RF and especially dialysis dependence are concerned with higher mortality, because the majority of patients requiring dialysis are unable to achieve renal recovery.^2^, ^5^, ^6^ Due to the little of clinical trials conducted in severe RF requiring dialysis in MM, the effect factors of reversal of renal failure and recovery from hemodialysis dependence are unknown.

In the current study, we examined clinical features and response to therapy in newly diagnosed MM (NDMM) patients with severe RF requiring dialysis who received initial therapy including novel agents to evaluate the promising factors of recovery from dialysis dependence and investigate the prognostic impact of dialysis independence for survival results.

## 2 MATERIALS AND METHODS

### 2.1 Patients and methods

This retrospective study included 45 NDMM patients with serious renal failure requiring hemodialysis between February 2009 and May 2017 at Tianjin Medical University General Hospital. Patients with light chain deposition disease, light chain (AL) amyloidosis or hemodialysis due to reasons rather than multiple myeloma were excluded from the study. The characteristics of patients are described in Table 1.

**Table 1:** Clinical and laboratory characteristics of 45 NDMM patients with severe renal failure requiring dialysis based on dialysis discontinuation or dependence after therapy

	All patients (n = 45)	Dialysis discontinuation after therapy (n = 22)	Dialysis dependence after therapy (n = 23)	*P*‐value
Age (y)	62 (44‐83)	61 (44‐78)	67 (45‐83)	.097
Male (N [%])	33 (73.33)	16 (72.73)	17 (73.91)	.928
eGFR (mL/min/1.73 m^2^)	7.25 (3.89‐14.86)	7.84 (4.60‐14.86)	6.76 (3.89‐11.51)	.041
Blood urea nitrogen (mmol/L)	23.8 (7.1‐49.9)	23.6 (9.8‐32.3)	24 (7.1‐49.9)	.653
Uric acid (μmol/L)	583 (270‐1186)	612 (309‐1186)	537 (270‐908)	.039
Calcium (mmol/L)	2.45 (1.92‐3.69)	2.53 (2.25‐3.35)	2.35 (1.92‐3.69)	.028
Hemoglobin (g/L)	82 (52‐131)	84 (52‐131)	77 (53‐100)	.037
Serum albumin (g/L)	33 (19‐45)	32.5 (19‐43)	33 (22‐45)	.631
Serum globulin (g/L)	37 (15‐96)	45.5 (15‐96)	32 (19‐88)	.297
Lactate dehydrogenase (U/L)	270 (120‐976)	256 (120‐976)	289 (138‐794)	.596
β_2_‐Microglobulin (mg/L)	18.3 (6.27‐32.04)	16.25 (6.27‐26.03)	19.4 (8‐32.04)	.010
Bone marrow plasma cells (%)	42 (10.5‐88)	41.25 (15‐88)	42 (10.5‐88)	.892
Involved free light chain (mg/L)	4356 (600‐2520)	2997 (600‐21000)	9100 (1070‐25208)	.003
Kidney disease history (mo)	2 (0.25‐24)	1 (0.25‐7)	6 (0.5‐24)	.003
24‐h urine volume examination (n [%])				
≥1000 mL	2 (4.44)	2 (9.09)	0	.032
400‐1000 mL	19 (42.22)	13 (59.09)	6 (26.09)	
100‐400 mL	16 (35.56)	5 (22.73)	11 (47.83)	
<100 mL	8 (17.78)	2 (9.09)	6 (26.09)	
24‐h proteinuria examination (n [%])				
(−)	5 (11.11)	5 (22.73)	0	.020
<1 g	19 (42.22)	11 (50)	8 (34.78)	
1‐3.5 g	10 (22.22)	2 (9.09)	8 (34.78)	
≥3.5 g	11 (24.44)	4 (18.18)	7 (30.43)	
Hematuria (n [%])	18 (40)	6 (27.27)	12 (52.17)	.130
Durie‐Salmon staging system (n [%])				
Ⅰ	0	0	0	.233
Ⅱ	2 (4.44)	2 (9.09)	0	
Ⅲ	43 (95.56)	20 (90.91)	23 (100)	
Revised International Staging System (n [%])				
Ⅰ	0	0	0	.069
Ⅱ	11 (24.44)	8 (36.36)	3 (13.04)	
Ⅲ	34 (75.56)	14 (63.64)	20 (86.96)	
M‐component type (n [%])				
IgG	14 (31.11)	7 (31.82)	7 (30.43)	.455
IgA	12 (26.67)	4 (18.18)	8 (34.78)	
Light chain only	19 (42.22)	11 (50)	8 (34.78)	
High‐risk FISH (n [%])	11 (24.44)	4 (18.18)	7 (30.43)	.339
Received novel agent (n [%])				
Bortezomib	32 (71.11)	16 (72.73)	16 (69.57)	.815
Thalidomide	13 (28.89)	6 (27.27)	7 (30.43)	
Treatment results (n [%])				
At least VGPR	18 (40)	15 (68.18)	3 (13.04)	<.001
Not up to VGPR	27 (60)	7 (31.82)	20 (86.96)	
Complication (n [%])				
Hypertension	22 (48.89)	7 (31.82)	15 (65.22)	.350
Diabetes	5 (11.1)	1 (4.55)	4 (17.39)	
Coronary disease	7 (15.56)	4 (18.18)	3 (13.04)	

Note

Kidney disease history: from the first time of the discovery of renal insufficiency symptoms until the time of initial treatment of myeloma.

Abbreviations: eGFR, estimated glomerular filtration rate; VGPR, very good partial response.

Renal function was assessed using the estimated glomerular filtration rate (eGFR), which was calculated by the simplified Modification of Diet in Renal Disease (MDRD) formula.^7^ Renal failure requiring hemodialysis was defined as an eGFR < 15 mL/min/1.73 m^2^ and on hemodialysis at initial diagnosis. In order to analyze data in our study, the patients were classified depending on renal function at diagnosis and response treatment: group 1: on dialysis at diagnosis improved to dialysis independence after therapy and group 2: on dialysis at diagnosis and remained dialysis dependence after treatment.

### 2.2 Treatment

All patients received chemotherapy as first‐line therapy and similar supportive care. Thirty‐two patients (71.11%) received bortezomib‐based combinations. The regimen included bortezomib and dexamethasone (VD) in 68.75% of patients; bortezomib, cyclophosphamide, and dexamethasone (VCD) in 25%; and bortezomib, melphalan, and prednisolone (VMP) in 6.25%. Thirteen patients (28.89%) received a thalidomide‐based therapy as first‐line chemotherapy. Therapy comprised thalidomide, melphalan and dexamethasone (MPT) in 69.23% of patients; thalidomide, cyclophosphamide and dexamethasone (CTD) in 15.38%; and dexamethasone and thalidomide (TD) in 15.38%.

The degree of recovery of renal function was estimated on the basis of the International Myeloma Working Group (IMWG) criteria:^2^, ^3^ A sustained improvement of baseline eGFR to ≥ 60 mL/min/1.73 m^2^ was defined as renal complete response (CRrenal), a sustained improvement of baseline eGFR from < 15 to 30‐59 mL/min/1.73 m^2^ as renal partial response (PRrenal) and a sustained improvement of baseline eGFR of <15 mL/min/1.73 m^2^ to 15‐29 mL/min/1.73 m^2^ or if baseline eGFR was 15‐29 mL/min/1.73 m^2^, improvement to 30‐59 mL/min/1.73 m^2^ as renal minor response (MRrenal). Treatment response was evaluated at the date of termination of each treatment according to the standard IMWG criteria.^8^ From the first day of treatment until the day of first confirmative, renal response (at least MRrenal) was estimated as time to renal response. From the first day of treatment until the day of first confirmative, maximum renal response was estimated as time to major renal response. From the first time of the discovery of renal insufficiency symptoms until the time of initial treatment of myeloma was defined as kidney disease history.

The time from diagnosis until date of death or date of last follow‐up was estimated as overall survival (OS). The time from treatment initiation until date of progression, death, or last follow‐up was estimated as progression‐free survival (PFS).

### 2.3 Statistical analysis

Mann‐Whitney *U* test was used for the comparisons for continuous variables among different groups. Pearson's chi‐square test was used for categorical variables, and Fisher's exact test was used when appropriate. The multivariate analysis was performed by logistic regression for factors of reversibility from dialysis dependence. Survival curves were plotted according to the Kaplan and Meier method, and comparisons among different groups were executed by the log‐rank test. With regard to multivariate analysis, factors associated with OS among NDMM patients with serious renal impairment requiring hemodialysis were introduced into a Cox proportional hazards model. Statistical analyses were performed using SPSS version 21.0 software. *P* values of <.05 were considered significant.

## 3 RESULTS

### 3.1 Patient characteristics

In our study, the median age was 62 years (range: 44‐83 years) and 15 patients were ≥70 years old at diagnosis. Thirty‐three patients (73.3%) were male. The median eGFR was 7.25 mL/min/1.73 m^2^ (range: 3.89‐14.86 mL/min/1.73 m^2^). The median calcium level was 2.45 mmol/L (range: 1.92‐3.69 mmol/L), and 10 patients (22.22%) had hypercalcemia. Median level of hemoglobin was 82 g/L (range: 52‐131 g/L) and 42 patients (93.33%) of hemoglobin level were ≤ 100 g/L. The median level of β_2_‐Microglobulin was 18.3 mg/L (range: 6.27‐32.04 mg/L). About 31.11% of all patients (14 patients) had MM of the IgG type, 26.67% had IgA type and 19 patients had light chain only type. The median involved free light chain (iFLC) level was 4356 mg/L (range: 600‐25208 mg/L). The median kidney disease history was 2 months (range: 0.25‐24 months). About 11.11% of all patients (five patients) were with proteinuria level of 3.5 g/24 h or higher, and 24 patients (68.57%) of urine volume examination were <400 mL/24 h. These indicators are the results of initial diagnosis (Table 1).

### 3.2 Response to chemotherapy

In the present study, at least very good partial response (VGPR) rate was 46.88% in patients received bortezomib‐based regimens, and 23.08% in those receiving thalidomide‐based regimens. The overall response rate was 78.13% in patients received bortezomib‐based combinations, and 76.92% in those treated with thalidomide‐based therapy (including CR, very good partial response, and partial response) (Table 2).

**Table 2:** Response to chemotherapy of 45 NDMM patients with severe renal failure requiring dialysis received bortezomib‐based regimens or thalidomide‐based regimens

	All patients (n = 45)	bortezomib‐based regimens (n = 32)	thalidomide‐based regimens (n = 13)
ORR (% [n])	77.78 (35)	78.13 (25)	76.92 (10)
At least VGPR (%[n])	40 (18)	46.88 (15)	23.08 (3)
CRrenal (%[n])	20 (9)	15.63 (5)	30.77 (4)
PRrenal (%[n])	22.22 (10)	25 (8)	15.38 (2)
MRrenal (%[n])	8.89 (4)	9.38 (3)	7.69 (1)
Hemodialysis independence (%[n])	48.89 (22)	50 (16)	46.15 (6)

Abbreviations: CRrenal, renal complete response; MRrenal, renal minor response; ORR, overall response rate (including CR, very good partial response, and partial response); PRrenal, renal partial response and VGPR, very good partial response.

**Figure 1:**
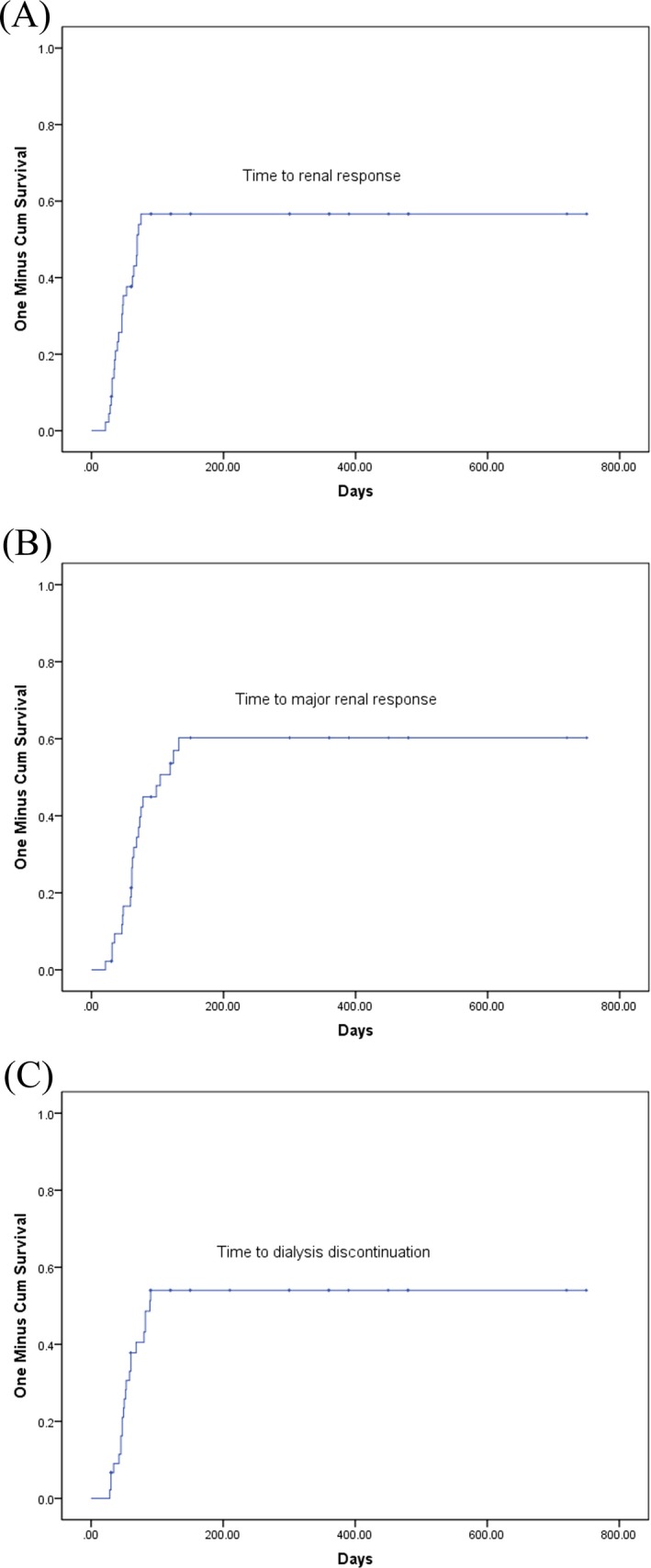
A, Time to renal response for 45 NDMM patients with severe renal failure requiring hemodialysis. B, Time to major renal response among 45 NDMM patients with severe renal failure requiring hemodialysis. C, Time to dialysis discontinuation for 45 NDMM patients with severe renal failure requiring hemodialysis

At least MRrenal was 51.11% in total patients. CRrenal was 20%, and PRrenal was 22.22% (major renal response rate was 42.22%) (Table 2). In addition, the median time to major renal response was 104 days (range: 21‐750 days). The median time to renal response was 69 days (range: 21‐750 days) (Figure 1 A,B).

Twenty‐two of the 45 (48.89%) cases were on hemodialysis at diagnosis and became hemodialysis independence, in which 16 patients (72.73%) received bortezomib‐based regimens and 6 patients (27.27%) received thalidomide‐based regimens. The median time to dialysis independence was 89 days (range: 28‐750 days) (Figure 1C).

Then we analyzed for factors that could be related to probability of dialysis discontinuation among NDMM patients with serious renal impairment requiring hemodialysis (Table 1). In the univariate analysis, the factors related to reversal of hemodialysis dependence were eGFR (*P* = .41), uric acid (*P* = .039), calcium (*P* = .028), hemoglobin (*P* = .037), β2‐Microglobulin (*P* = .010), involved free light chain (*P* = .003), kidney disease history (*P* = .003), 24‐hour urine volume (*P* = .032), 24‐hour proteinuria (*P* = .020), and achieving at least VGPR (*P* < .001). In the logistic regression analysis, kidney disease history (OR 1.667, 95% CI 1.133‐2.452, *P* = .009), β2‐Microglobulin (OR 1.412, 95% CI 1.075‐1.856, *P* = .013), involved free light chain (OR 1.461, 95% CI 1.127‐1.895, *P* = .004), and achieving at least VGPR (OR 0.394, 95% CI 0.179‐0.870, *P* = .021) were significantly associated with reversibility from dialysis dependence (Table 3).

**Table 3:** Multivariate logistic regression analysis of factors associated with reversibility from dialysis dependence

	Odds ratio (95% CI)	*P*
Kidney disease history	1.667 (1.133‐2.452)	.009
β2‐Microglobulin	1.412 (1.075‐1.856)	.013
Involved free light chain	1.461 (1.127‐1.895)	.004
Achieving at least VGPR	0.394 (0.179‐0.870)	.021

Abbreviations: Kidney disease history, from the first time of the discovery of renal insufficiency symptoms until the time of initial treatment of myeloma; VGPR, very good partial response.

### 3.3 Survival outcomes

Early death occurred in 4 (8.89%) cases (within 2 months from treatment initiation). The median follow‐up time was 12 months (range, 1‐48 months). The median OS was 13 months (95% CI 4.326‐21.674). The following variables were analyzed to identify factors acting on OS in the Table 1 among NDMM patients with severe renal failure requiring hemodialysis: age gender, eGFR, blood urea nitrogen (BUN), uric acid (UA), calcium, hemoglobin, serum albumin, serum globulin, lactate dehydrogenase (LDH), β2‐Microglobulin, bone marrow plasma cells, involved free light chain, kidney disease history, 24‐hour urine volume, 24‐hour proteinuria, hematuria, Durie‐Salmon (DS) staging system, Revised International Staging System (R‐ISS), M‐component type, high‐risk FISH, treatment with bortezomib, reversibility from dialysis dependence, achieving at least VGPR, and complication. The multivariate analyses demonstrated that reversibility from dialysis dependence (HR 0.316, 95% CI 0.128‐0.777, *P* = .012), proteinuria < 3.5 g/24 h (HR 0.258, 95% CI 0.072‐0.925, *P* = .038), and achieving at least VGPR (HR 0.226, 95% CI 0.082‐0.625, *P* = .004) were significantly associated with OS among NDMM patients with severe renal failure requiring hemodialysis (Table 4).

**Table 4:** Multivariate analysis of factors associated with overall survival among NDMM patients with severe renal failure requiring hemodialysis

	Hazard ratio (95% CI)	*P*
Reversibility from dialysis dependence	0.316 (0.128‐0.777)	.012
Proteinuria < 3.5 g/24 h	0.258 (0.072‐0.925)	.038
Achieving at least VGPR	0.226 (0.082‐0.625)	.004

Abbreviation: VGPR, very good partial response.

**Figure 2:**
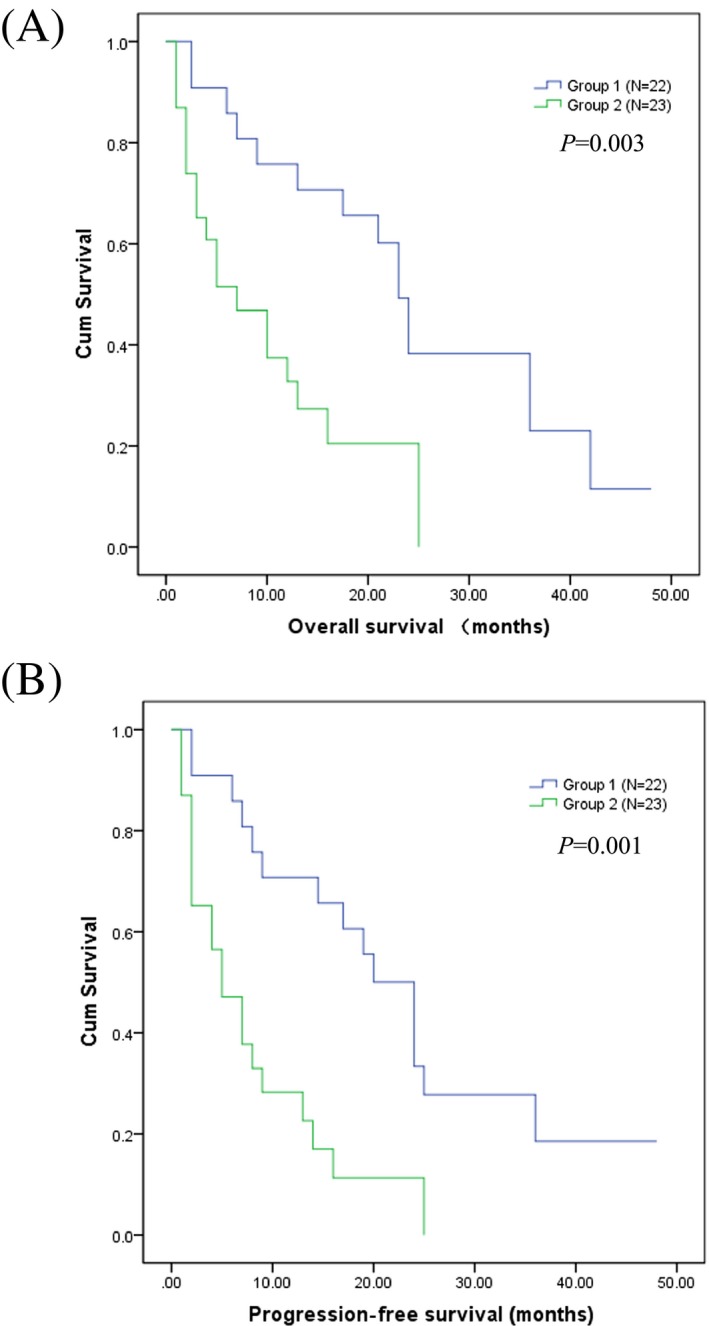
Kaplan‐Meier plot comparing (A) overall survival and (B) progression‐free survival between groups 1 and 2 based on their renal function at diagnosis and response to therapy: group 1, on dialysis at diagnosis improved to dialysis discontinuation after therapy; group 2, on dialysis at diagnosis and remained dialysis dependence after therapy

Upon categorizations were on the basis of renal function at diagnosis and response to treatment. After comparison, the median OS of group 1 (N = 22) was 23 months and group 2 (N = 23) was 7 months (*P* = .003). In addition, the median PFS of group 1 was 20 months and group 2 was 5 months (*P* = .001) (Figure 2 A,B).

## 4 DISCUSSION

Multiple myeloma (MM) is a malignancy of malignant plasma cells and occurs renal insufficiency in 20%‐50% of patients.^5^, ^9^ Up to 10% of MM patients can present with serious renal impairment requiring dialysis.^10^ Renal failure is related to a growth risk of early death and treatment‐related toxicity.^2^ Many studies have confirmed that renal failure and reversal of hemodialysis dependence can lead to enormous survival benefits.^5^, ^11^, ^12^ However, the effect factors of recovery from renal impairment and reversal of hemodialysis dependence are unknown. Our study investigated that shorter kidney disease history, lower serum level of β2‐microglobulin and free light chain, and achieving at least VGPR were significantly associated with reversibility from hemodialysis dependence and reaching hemodialysis discontinuation was related to better survival.

Dimopoulos et al suggests that three drugs, VD‐based regimen are related to a high probability of renal response and with 50% probability of hemodialysis independency in MM patients requiring hemodialysis, without the use of plasmapheresis or HCO filters.^13^ However, in our study, 48.89% achieved probability of hemodialysis dependence in NDMM patients who require hemodialysis at diagnose. Sixteen patients (72.73%) received bortezomib‐based regimens and six patients (27.27%) received thalidomide‐based regimens. But, sixteen patients (69.57%) received bortezomib‐based regimens and seven patients (30.43%) received thalidomide‐based regimens among patients remained dialysis dependence after therapy. Treatment with bortezomib‐based regimens may not be a direct factor for recovery from dialysis dependence.

Many studies have confirmed that bortezomib‐based therapy was very effective for the treatment of MM patients with renal failure^14^, ^15^, ^16^, ^17^, ^18^ and could reverse dialysis dependence.^10^, ^19^ In our study, at least VGPR rate was 46.88% in patients received bortezomib‐based therapy, and 23.08% for those treated with thalidomide‐based combinations. But, the overall response rate was 78.13% in patients received bortezomib‐based therapy, and 76.92% for those treated with thalidomide‐based combinations. Using bortezomib‐based regimens improved at least VGPR rate distinctly. In addition, achieving at least VGPR (OR 0.394, 95% CI 0.179‐0.870, *P* = .021) was significantly related to reversibility from dialysis dependence. In summary, bortezomib‐based regimens were strongly recommended for treatment of MM patients requiring dialysis.

In the meanwhile, the other study from the group of Dimopoulos has recently investigated the outcomes of 52 NDMM patients requiring dialysis.^12^ Early mortality (within 2 months from start of therapy) was 16%, in most cases because of infectious complications. In our study, early death occurred in 4 (8.89%) cases on account of infection and heart failure. Early mortality owing to MM with severe renal failure is inevitable, but it may represent a serious confounding effect for the purpose of the planned analysis.^2^, ^3^ Furthermore, they suggested that twenty‐six (50%) patients achieved a renal response and became dialysis independent. Age ≤ 65 years (*P* = .027) and free light chain levels ≥ 9000 mg/L (*P* = .1) were related to higher probability to renal response. MM patients who achieved at least PR within the first 2 months had higher rates of dialysis independence (*P* = .004). However, their results were only based on univariate analysis, and we not only did univariate analysis, but also analyzed with multivariate factors. In addition to the discovery of involved free light chain and achieving at least VGPR, we also found that β2‐Microglobulin and renal kidney disease history were associated with higher probability to dialysis independence.

The history of kidney disease affected probability of dialysis independence, which displayed that patients who are discovered with the following situations should be suspected to be myeloma immediately and treated aggressively: (a) age > 40 with unknown cause of renal insufficiency; (b) the degree of anemia and renal damage is not proportional; (c) nephritic syndrome without hematuria, hypertension, or early stage of kidney disease with anemia and renal failure; (d) renal insufficiency with hypercalcemia; (e) increased erythrocyte sedimentation rate, hypergammaglobulinemia and infection (urinary tract and respiratory tract); and (f) inconsistent results of proteinuria by urine routine test and 24‐hour urine protein test.^20^, ^21^ It is essential to be able to recognize the symptoms of myeloma in clinical routine and to be aware of basic diagnostic features to confirm this disease and give timely treatment.^21^, ^22^, ^23^, ^24^

In addition, β_2_‐microglobulin (β_2_M) is expressed on the surface of all nucleated cell and associated with the major histocompatibility complex I (MHC‐I)/human leukocyte antigen I (HLA‐I) to facilitate antigen presentation.^25^ β_2_M, which has been introduced into staging system of MM and thought to a reflection of renal function and the tumor cell burden, has been regarded as the most important prognostic factor in MM.^26^, ^27^ Recent studies discovered that β_2_M is not only a biomarker in patients with chronic kidney disease and end‐stage renal disease, but also a promising marker to assess glomerular and tubular function in adults.^28^ However, our study first found the role of β_2_M in the reversal of renal failure and recovery from dialysis dependence.

It is known to us that serum FLC assays in diagnostic screening panels were recommended currently by International Myeloma Working Group (IMWG).^29^ In addition, Thomas et al illustrated that serum free light chains should be used to evaluate response in light chain multiple myeloma.^30^ However, our study found the prognostic value of involved free light chain which was significantly related to reversibility from dialysis dependence. This is possibly associated with the monoclonal light chains on basement membranes of the glomeruli and/or the renal tubule which cause the reason of MM patients with renal impairment.^3^, ^31^ In the meantime, Dimopoulos *et al* suggest that very high levels of FLCs were associated with a lower probability of RF recovery or dialysis discontinuation.^12^, ^13^

In a conclusion, our data indicate that the shorter kidney disease history, lower serum level of β2‐microglobulin and free light chain, achieving at least VGPR is related to a high probability of hemodialysis discontinuation and bortezomib‐based regimens were strongly recommended for treatment of MM patients requiring dialysis. In addition, recovery from dialysis dependence, proteinuria <3.5 g/24 h, and achieving at least VGPR can lead to enormous survival benefits in NDMM patients with serious renal impairment requiring hemodialysis. Our study offered instructions for treatment and evaluated prognosis of NDMM patients with severe renal failure requiring dialysis.

## AUTHORS' CONTRIBUTIONS

RF designed the research and revised the manuscript. JS, FJ, and HL analyzed the data and wrote the article. KD, YR, LL, GW, and ZS contributed to the collection of the patients^,^ data. All authors read and approved the final manuscript.
